# Global trends and knowledge structure of tetanus research from 2005 to 2025: A bibliometric analysis based on web of science Core Collection and Scopus

**DOI:** 10.1016/j.onehlt.2026.101590

**Published:** 2026-09-20

**Authors:** Dandan Zhou, Ji Zhang, Ke Ma, Tianjing Sun, Fangfang Deng, Shengping Wu, Han Hu, Haizhen Duan, Tianxi Zhang, Anyong Yu, Xuheng Jiang

**Affiliations:** a Department of Emergency, Affiliated Hospital of Zunyi Medical University, Zunyi, Guizhou, China; b Department of Infectious Diseases, Affiliated Hospital of Zunyi Medical University, Zunyi, Guizhou, China

**Keywords:** Tetanus, Bibliometric analysis, Immunization, Maternal and neonatal tetanus, Vaccine-preventable diseases

## Abstract

**Background:**

Tetanus remains a vaccine-preventable but potentially fatal disease, with control relying on vaccination coverage, maternal/neonatal protection, adult boosters, wound prophylaxis, and clinical management. This study characterized the global research landscape, thematic evolution, and emerging frontiers of tetanus literature from 2005 to 2025.

**Methods:**

English-language articles and reviews were retrieved from the Web of Science Core Collection (WoSCC) and Scopus, with the final search conducted on May 12, 2026. Records were merged, deduplicated, and manually screened. Bibliometrix, VOSviewer, CiteSpace, and Scimago Graphica were used for descriptive analyses and visualization of collaboration, keyword co-occurrence, co-citation, and burst patterns. Citation-trend and reference co-citation analyses were restricted to WoSCC records. A manually verified clinical-treatment subset underwent supplementary keyword analysis.

**Results:**

Of 28,912 retrieved records, 8307 publications met the inclusion criteria, comprising 7689 articles and 618 reviews. Annual publication output remained relatively stable, with a modest increase after 2010. The United States contributed the most publications (2578; 31.03%), followed by the United Kingdom, India, Germany, and China. The Centers for Disease Control and Prevention was the most productive institution, and Vaccine was the leading journal (643 publications). Three broad thematic domains emerged: toxin biology and immune mechanisms, vaccines and immunization strategies, and public health implementation and population protection. Recent keyword bursts highlighted vaccination coverage, pregnancy, women, vaccine hesitancy, and public health, while mechanistic and immunological research remained active. The clinical-treatment subset included 675 publications and emphasized intensive care, antitoxin and immunoglobulin therapy, autonomic dysfunction, and mortality.

**Conclusions:**

Recent tetanus research fronts increasingly emphasize immunization delivery and population protection, alongside continuing mechanistic research. Clinical management constitutes a distinct but less prominent subdomain. These findings provide a publication-level overview to inform future evidence synthesis and research planning but should be interpreted alongside clinical, epidemiological, and surveillance evidence.

## Highlights

• Tetanus publication output remained broadly stable during 2005–2025.
• High-income countries showed high output and strong international collaboration.
• Mechanistic research persisted alongside growing implementation-oriented vaccine research.
• Maternal immunization and booster vaccination gained recent bibliometric prominence.
• Clinical management remained an important treatment-related research domain.

## 1 Introduction

Tetanus is an acute, life-threatening neurological disease caused by *Clostridium tetani*, an anaerobic, spore-forming bacterium that produces tetanospasmin, one of the most potent bacterial neurotoxins known [1]. Following entry through contaminated wounds, burns, umbilical stumps, or other breaches in the skin or mucosa, *C. tetani* spores may germinate under anaerobic conditions and release tetanus toxin. By blocking the release of gamma-aminobutyric acid and glycine, the toxin impairs inhibitory neurotransmission, causing muscle rigidity, painful spasms, autonomic instability, respiratory compromise, and potentially death [2], [3]. Although vaccine-preventable, tetanus remains clinically important because natural infection does not reliably confer immunity, and sustained protection requires primary immunization and timely boosters [1], [4].

Over the past several decades, global immunization programs have substantially reduced the incidence and mortality of tetanus, especially neonatal and maternal tetanus [2], [5]. The introduction and expansion of tetanus toxoid-containing vaccines, antenatal immunization, clean delivery practices, improved wound management, and post-exposure prophylaxis have played central roles in tetanus prevention [1]. Nevertheless, tetanus has not been eradicated because *C. tetani* spores are widely distributed in the environment, and disease elimination depends on maintaining high vaccination coverage rather than interrupting transmission [1]. In many low- and middle-income countries, incomplete immunization, limited access to healthcare, unsafe delivery conditions, and delayed treatment continue to contribute to preventable tetanus morbidity and mortality [5], [6]. In high-income countries, tetanus has become rare but still occurs among older adults, individuals with incomplete vaccination histories, people who inject drugs, and patients with inadequate wound prophylaxis [3], [4]. Therefore, tetanus remains a relevant topic for infectious disease control, vaccine policy, emergency medicine, maternal and child health, and global public health.

Previous studies on tetanus have mainly focused on clinical manifestations, intensive care management, vaccine effectiveness, maternal and neonatal tetanus elimination, immunization strategies, and epidemiological burden [2], [3], [7]. However, tetanus research spans microbiology, neurology, pediatrics, obstetrics, immunology, vaccinology, public health, and global health. Its overall knowledge structure, collaboration networks, thematic evolution, and research hotspots therefore remain insufficiently summarized quantitatively and visually. Although narrative and clinical reviews effectively synthesize specific topics, they are less suited to mapping large bodies of literature, identifying influential contributors, tracing intellectual structures, and detecting emerging research fronts [8].

Bibliometric analysis is widely used to quantify scientific output, collaboration patterns, citation impact, research hotspots, and knowledge evolution [8], [9]. Visualization tools such as CiteSpace, VOSviewer, and Bibliometrix can further reveal co-authorship networks, keyword structures, co-citation patterns, and emerging trends. Combining Web of Science Core Collection (WoSCC) and Scopus also helps improve literature coverage and reduce database-related bias [10].

Although a recent bibliometric and integrative review has examined global research on tetanus vaccines and tetanus immunoglobulins [11], the broader tetanus research landscape remains insufficiently characterized. This study therefore analyzed tetanus-related publications indexed in WoSCC and Scopus from 2005 to 2025, focusing on publication and citation trends, leading contributors, collaboration networks, research hotspots, and intellectual structures. The findings provide a systematic overview to inform future clinical research and public health decision-making.

## 2 Materials and methods

### 2.1 Literature search and data collection

This bibliometric study characterized publication trends, collaboration patterns, intellectual structure, and research hotspots in global tetanus-related research from 2005 to 2025. Bibliographic records were retrieved from the Web of Science Core Collection and Scopus. The two databases were jointly used to reduce potential bias arising from differences in journal coverage, indexing policies, and metadata structures [8], [10].

WoSCC was searched using the Topic field, whereas Scopus was searched using the TITLE-ABS-KEY field. The search terms covered tetanus, *Clostridium tetani*, tetanus toxin and toxoid, vaccination, immunoglobulin, prophylaxis, maternal and neonatal tetanus, and major clinical forms of tetanus.

For Web of Science Core Collection: TS = (“tetanus” OR “*Clostridium tetani*” OR “*C. tetani*” OR “C tetani” OR “tetanospasmin” OR “tetanus toxin” OR “tetanus toxoid*” OR “tetanus immunoglobulin” OR “tetanus antitoxin” OR “tetanus vaccine” OR “tetanus immunization” OR “tetanus prophylaxis” OR “tetanus booster” OR “neonatal tetanus” OR “maternal tetanus” OR “obstetric tetanus” OR “maternal and neonatal tetanus” OR “maternal‑neonatal tetanus” OR “cephalic tetanus” OR “generalized tetanus” OR “local tetanus” OR “localized tetanus” OR “post-traumatic tetanus” OR “posttraumatic tetanus” OR “traumatic tetanus”).

The same terms were applied to the TITLE-ABS-KEY field in Scopus. Eligible publications were English-language articles and reviews published between January 1, 2005, and December 31, 2025. The final search was conducted on May 12, 2026. Reviews were retained because they synthesize existing knowledge and contribute to citation, co-citation, and keyword networks; however, their inclusion may influence citation-based indicators because reviews are often highly cited. Other document types were excluded because of their generally limited reference metadata and suitability for bibliometric analysis.

Full bibliographic records and cited references were exported, including titles, authors, abstracts, keywords, journals, publication years, affiliations, and citation counts. Retrieval and screening counts are reported in Section 3.1 and Fig. 1A.

**Fig. 1:**
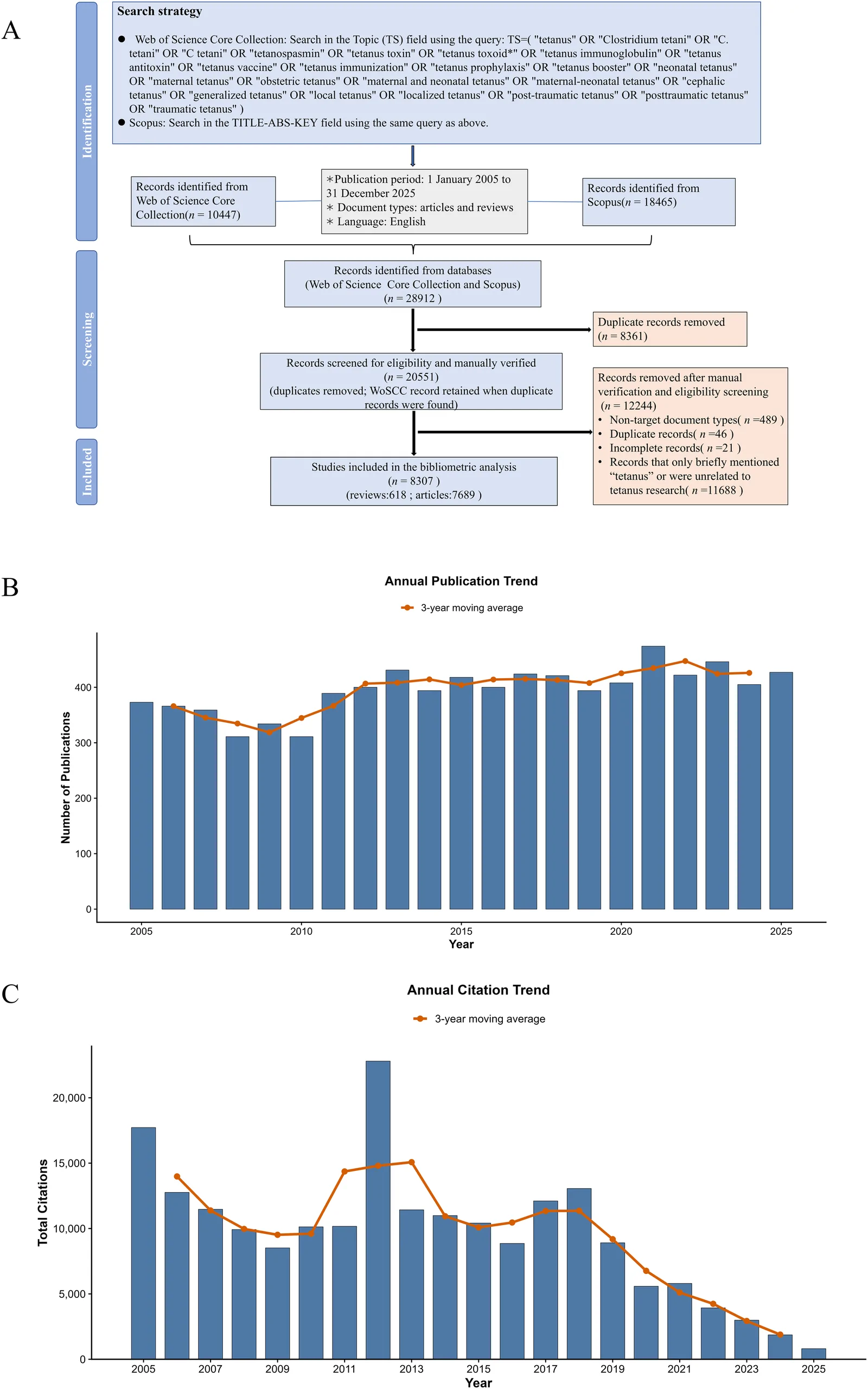
Literature selection and temporal trends of tetanus-related research. A. Literature search and screening process. B. Annual publication trend. C. Annual citation trend based on WoSCC.

### 2.2 Data cleaning and inclusion criteria

Records were imported into R and merged using DOI as the primary deduplication identifier. DOI strings were lowercased, and URL prefixes, extra spaces, and trailing punctuation were removed. Records without valid DOIs were matched using normalized title, first-author surname, and publication year. Titles were normalized through lowercasing, punctuation removal, whitespace compression, and special-character standardization. For publications indexed in both databases, the WoSCC record was retained. The final dataset comprised 8307 publications: 6978 WoSCC records and 1329 Scopus-only records.

Country/region labels extracted from affiliations were harmonized using a manually curated lookup table (Supplementary Table S1). No dataset-wide disambiguation or standardization was performed for author names, institutional aliases, journal titles, or cited-reference strings.

An R script was used only to flag and organize potentially ineligible records according to document type and metadata completeness; it did not make any inclusion or exclusion decisions. Apart from primary algorithmic deduplication, all removal and eligibility decisions were made manually using titles, abstracts, and available author keywords. Residual duplicates, which could not be identified automatically due to inconsistent cross-database metadata, were removed during manual verification. Remaining records were assessed against hierarchical, mutually-exclusive exclusion criteria; each excluded record was assigned one primary exclusion reason: (1) non-target document type; (2) insufficient bibliographic metadata for relevance assessment; (3) no direct relevance to tetanus research; (4) residual duplicate identified during manual review.

Publications were considered relevant when their principal focus was tetanus, *C. tetani*, tetanus toxin or pathogenesis, or tetanus-related vaccination, seroprotection, clinical management, prophylaxis, maternal or neonatal disease, epidemiology, or disease burden. Records were excluded if tetanus appeared only as background information, in a comparator or medical-history context, or if tetanus toxoid was used solely as a carrier protein for another vaccine.

One researcher performed all screening and reassessed records with uncertain eligibility.

A supplementary clinical-treatment subset was identified from the final dataset without an additional database search. Candidate records were flagged by matching titles, abstracts, and author keywords against the following Boolean term set: “treatment” OR “therapy” OR “clinical management” OR “intensive care” OR “ICU” OR “critical care” OR “mechanical ventilation” OR “ventilation” OR “airway” OR “sedation” OR “benzodiazepine” OR “magnesium sulfate” OR “magnesium sulphate” OR “muscle spasm” OR “spasm control” OR “autonomic dysfunction” OR “autonomic instability” OR “tetanus immune globulin” OR “tetanus immunoglobulin” OR “TIG” OR “antitoxin” OR “immunoglobulin” OR “wound management” OR “wound care” OR “debridement” OR “post-exposure prophylaxis” OR “prophylaxis”. Automated term matching was used only to identify candidate records because several terms may refer to either prevention or treatment.

Candidates were manually reviewed, retaining only publications addressing treatment of patients with established tetanus, including intensive care, antitoxin therapy, symptom control, and complication management. Prevention-only studies were excluded, and uncertain records were reassessed by the same researcher. This subset was used exclusively for supplementary keyword co-occurrence analysis and did not alter the main dataset.

### 2.3 Bibliometric and statistical analyses

Bibliometric and visual analyses were performed using VOSviewer (version 1.6.20), CiteSpace (version 6.4.1), Scimago Graphica, and the Bibliometrix package in R (version 4.4.1) [9], [12], [13]. Unless otherwise specified, analyses used the merged, deduplicated dataset. Citation-trend and reference co-citation analyses were restricted to the 6978 eligible WoSCC records.

Country-level analyses used full counting, assigning each publication to every country/region represented in its affiliations. Publications with affiliations from at least two distinct countries/regions were classified as multi-country publications. For each country/region, MCP% was calculated as the number of multi-country publications divided by its total publication count, multiplied by 100.

Bibliometrix was used to summarize annual publication output and country-level collaboration. Publication and citation trends were displayed as bar charts with centered three-year moving averages. Citation totals were calculated by summing the cumulative WoSCC citation counts, recorded on the search date, of eligible publications from each publication year.

VOSviewer constructed author- and institution-level collaboration networks under full-counting mode with clustering resolution set to 1.0. Association-strength normalization was applied for link-strength normalization. Network-specific minimum occurrence thresholds are reported in corresponding figure captions. Total link strength follows the native VOSviewer definition: the sum of link strengths connecting a given node to all other nodes. In country-co-authorship networks, link strength between two countries equals the number of jointly authored publications under full counting. Total link strength aggregates all pairwise collaborative links for a node and does not directly equal the raw count of multi-country publications, as a single multilateral publication contributes to multiple pairwise links [12]. Geographic collaboration maps and ring charts were generated using Scimago Graphica.

CiteSpace was used for keyword co-occurrence, clustering, and burst analyses; cited-journal and journal dual-map analyses; and reference co-citation, clustering, and burst analyses [13]. The analysis period was divided into one-year time slices from 2005 to 2025. Nodes were selected using the g-index with k = 8, except for the co-cited-journal network, for which the top 30 nodes per time slice were selected. Pathfinder pruning was applied to simplify network structures, and cluster labels were generated using the log-likelihood ratio algorithm. Keyword and reference bursts were detected using Kleinberg's algorithm with γ = 1.0 and a minimum duration of two consecutive years.

CiteSpace keyword analyses used author keywords (DE) and Keywords Plus (ID) for WoSCC records, and author keywords only for Scopus-only records. No additional keyword normalization, synonym unification, or singular–plural merging was performed before these analyses. R-based keyword normalization was applied only to the three-field Sankey diagram.

A three-field Sankey plot was generated using Bibliometrix to visualize relationships among countries/regions, source journals, and keywords. Node width represented publication volume or occurrence frequency, whereas link width represented association strength.

Outputs included publication counts, percentages, rankings, citation counts, average citations per publication, node frequencies, total link strength, and burst strength and duration. All analyses were descriptive, with no inferential statistical testing or causal inference. Citation-based indicators were interpreted in light of database-specific coverage and citation practices.

This study used bibliographic metadata from published literature and involved no human participants, private personal information, or biological specimens; ethical approval and informed consent were therefore not required.

## 3 Results

### 3.1 Literature selection and temporal trends

A total of 28,912 records were initially retrieved, including 10,447 from the Web of Science Core Collection and 18,465 from Scopus. Algorithmic deduplication removed 8361 records, leaving 20,551 provisionally unique records for eligibility assessment. During manual eligibility screening, 12,244 records were excluded: 489 were classified as non-target document types, 21 lacked sufficient bibliographic metadata for relevance assessment, 11,688 were not directly related to tetanus research, and 46 were residual duplicates identified during manual verification. The final bibliometric dataset comprised 8307 publications, including 7689 articles and 618 reviews. The literature selection process is summarized in Fig. 1A. Annual publication output remained relatively stable throughout 2005–2025, indicating sustained research activity in tetanus-related fields. A modest upward tendency was observed after 2010. Annual citation counts peaked around 2012 and declined in more recent years. This decline should be interpreted cautiously because recent publications have had less time to accumulate citations and records from the latest years may also be affected by database indexing delays (Fig. 1B–C).

### 3.2 Leading contributors and collaboration patterns

Within this English-language WoSCC-Scopus dataset, the global distribution of tetanus-related research showed clear concentration among several major contributing countries. As summarized in Table 1 and visualized in Fig. 2A-B, the United States ranked first with 2578 publications, accounting for 31.03% of all included records. It was followed by the United Kingdom with 895 publications, India with 656, Germany with 494, and China with 455. These countries represented the core contributors to tetanus-related research during the study period.

**Table 1:** Leading contributors to tetanus-related research from 2005 to 2025.

Panel A. Top productive countries				
Rank	Country	Documents	Documents %	MCP %
1	USA	2578	31.03	37.7
2	United Kingdom	895	10.77	65.03
3	India	656	7.9	28.35
4	Germany	494	5.95	52.83
5	China	455	5.48	30.55
6	France	418	5.03	66.03
7	Italy	372	4.48	46.51
8	Canada	358	4.31	62.01
9	Belgium	325	3.91	82.15
10	Australia	311	3.74	55.63


Panel B. Top productive authors				
Rank	Author	Documents	Citations	Total link strength
1	Nicola P. Klein	37	1969	31
2	Scott A. Halperin	34	982	15
3	Ray Borrow	29	937	14
4	Jacqueline M. Miller	28	569	60
5	Elisabeth A. M. Sanders	28	707	39
6	Guy A. M. Berbers	27	993	31
7	Pierre Van Damme	27	990	36
8	Marie Van der Wielen	26	476	42
9	Ornella Rossetto	23	719	40
10	Peng-Jun Lu	22	2052	16


Panel C. Top productive organizations				
Rank	Organization	Documents	Citations	Total link strength
1	Centers for Disease Control and Prevention	185	6730	85
2	GSK / GlaxoSmithKline	143	3879	33
3	University of Oxford	119	3531	38
4	Harvard University	92	5348	49
5	London School of Hygiene & Tropical Medicine	86	10300	49
6	Sanofi Pasteur	85	1490	16
7	University of Washington	82	4065	35
8	WHO	74	8234	48
9	The University of Melbourne	69	1719	39
10	Emory University	66	2635	33


Panel D. Top productive journals				
Rank	Journal	Documents	JIF(2025)	JCR(2025)
1	Vaccine	643	3.5	Q2
2	Human Vaccines & Immunotherapeutics	175	3.5	Q2
3	PLOS ONE	145	2.6	Q2
4	Vaccines	121	3.4	Q2
5	The Pediatric Infectious Disease Journal	115	2.2	Q2
6	Pediatrics	70	6.1	Q1
7	Frontiers in Immunology	67	5.9	Q1
8	The Journal of Immunology	58	3.4	Q2
9	Clinical Infectious Diseases	53	7.3	Q1
10	BMC Public Health	53	3.6	Q1

Note: MCP%, multi-country publication percentage, calculated for each country/region as the percentage of its publications involving affiliations from at least two distinct standardized countries/regions. Country/region publication counts were based on full counting using all author affiliations.

**Fig. 2:**
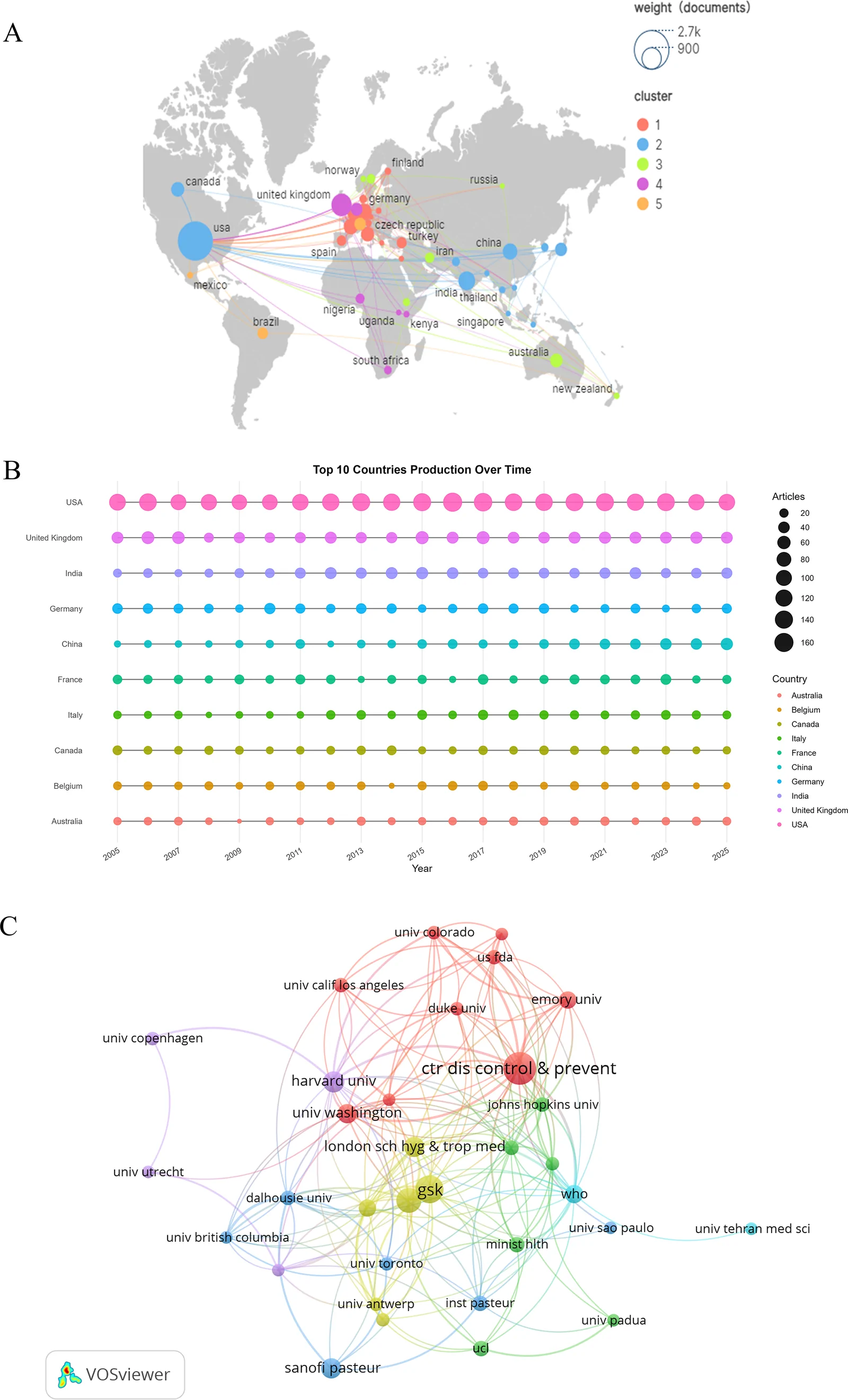
Global productivity and collaboration patterns in tetanus-related research. A. Geographical distribution and international collaboration network(minimum country occurrence = 50). B. Publication output of the top 10 countries over time. C. Institutional collaboration network(minimum documents per institution = 40).

The proportion of internationally co-authored publications varied among the leading countries/regions. As shown in Table 1, Belgium had the highest MCP% among the listed countries/regions (82.15%), followed by France (66.03%), the United Kingdom (65.03%), and Canada (62.01%). These percentages indicate that internationally co-authored publications accounted for relatively large shares of the output attributed to these countries/regions. The country/region collaboration network is presented in Supplementary Fig. S1, where the United States showed extensive collaborative links with multiple countries/regions.

Institutional analysis showed that public health agencies, universities, research institutes, and vaccine-related organizations were major contributors. The Centers for Disease Control and Prevention was the most productive institution and occupied a central position in the institutional collaboration network. GSK (GlaxoSmithKline), the London School of Hygiene & Tropical Medicine, Oxford, Harvard, and other academic institutions also showed strong research influence. As shown in Fig. 2C, institutional collaboration was more centralized than author-level collaboration, with public health agencies, universities, and vaccine manufacturers forming major collaborative hubs.

At the journal level, tetanus-related studies were mainly published in vaccine, infectious disease, pediatric, and public health journals. Vaccine was the most productive journal, publishing 643 articles, followed by Human Vaccines & Immunotherapeutics with 175, PLOS ONE with 145, Vaccines with 121, and The Pediatric Infectious Disease Journal with 115. The relationships among leading countries, source journals, and high-frequency keywords are further visualized in Supplementary Fig. S6, which highlights the central role of vaccine-related journals and immunization-oriented topics in the tetanus research landscape. Author-level collaboration was relatively fragmented, with multiple research clusters rather than a single dominant network. This pattern is further illustrated in Supplementary Fig. S2, which shows several author clusters connected by cross-group collaborations but no single overwhelmingly dominant author network.

### 3.3 Knowledge base and thematic structure

Reference co-citation and keyword co-occurrence analyses were used to characterize the knowledge base and thematic organization of tetanus research. As shown in Fig. 3A-B, the co-cited references and keyword clusters indicated that tetanus research was structured around several major domains rather than a single homogeneous topic. The underlying network structures are further illustrated in Supplementary Fig. S3 for co-cited references and Supplementary Fig. S4 for keyword co-occurrence, while journal-level intellectual structure and disciplinary citation patterns are presented in Supplementary Fig. S5

The first domain was related to mechanisms and basic immunity, including tetanus neurotoxin, cellular immunity, antibody response, immune activation, immunological memory, and toxin-related biological mechanisms. These topics represented the mechanistic foundation of tetanus research and provided the basis for understanding pathogenesis, immune protection, and vaccine-induced responses.

**Fig. 3:**
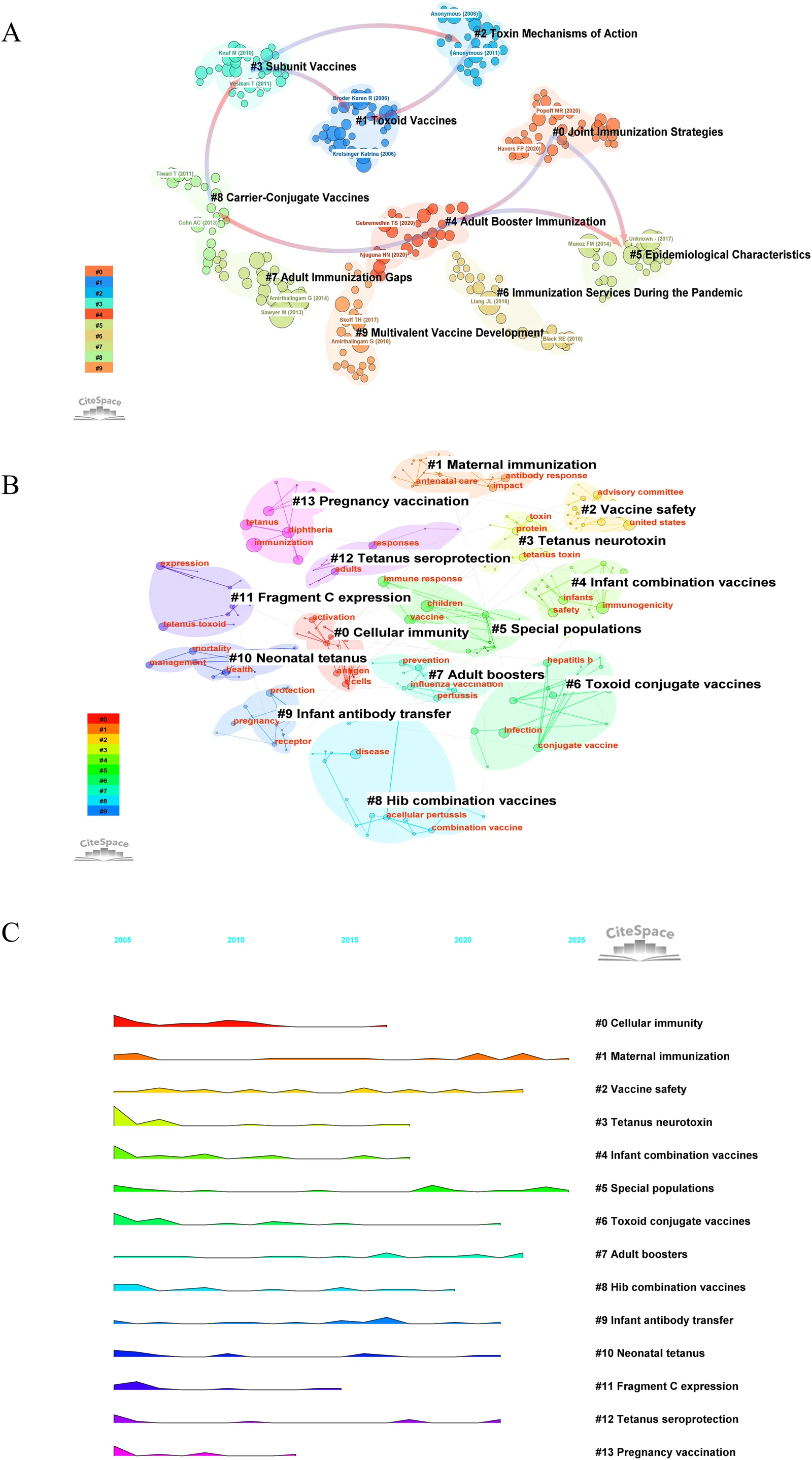
Knowledge base and thematic evolution of tetanus-related research. Co-cited reference clusters. B. Keyword clusters. C. Temporal evolution of keyword clusters.

The second domain was centered on vaccines and immunization strategies. This included tetanus toxoid vaccines, infant combination vaccines, diphtheria-tetanus-pertussis-containing vaccines, toxoid conjugate vaccines, maternal immunization, adult booster vaccination, reactogenicity, and vaccine safety. This domain reflected the central role of tetanus toxoid-containing vaccines in both childhood immunization programs and life-course vaccination strategies, consistent with the dense vaccine- and immunization-related nodes observed in Supplementary Fig. S4.

The third domain was related to public health implementation and population protection. Keywords in this domain included special populations, seroprotection, vaccination coverage, immunization services, maternal and neonatal tetanus, and vaccination during public health disruptions such as the COVID-19 pandemic. These clusters indicate that tetanus research has increasingly addressed population-level immunity, vulnerable groups, service delivery, and programmatic challenges.

Overall, the thematic structure demonstrates that tetanus research has been built on a continuum from toxin biology and immune mechanisms to vaccine development, immunization policy, and public health implementation. This multidimensional structure is also supported by the journal citation flows shown in Supplementary Fig. S5.

### 3.4 Thematic evolution and research frontiers

The temporal evolution of keyword clusters and burst analyses illustrated shifts in the focus of emerging tetanus-related research frontiers over the past two decades. Notably, mechanistic and immunology-oriented themes persisted across the full study timeframe, alongside newly emerging public-health-relevant research hotspots. As presented in Fig. 3C and Fig. 4A-B, early-stage burst keywords centered on immunological mechanisms and vaccine response research. Representative early burst terms included dendritic cells (strength = 17.32, 2005–2010), T cells (strength = 13.03, 2005–2011), antibody response (strength = 15.42, 2005–2007), memory (strength = 11.24, 2005–2008), and reactogenicity (strength = 16.69, 2006–2013). These burst metrics reflect prominent scholarly interest in cellular- and humoral-immune mechanisms underlying tetanus vaccine-induced protection during 2005–2013.

**Fig. 4:**
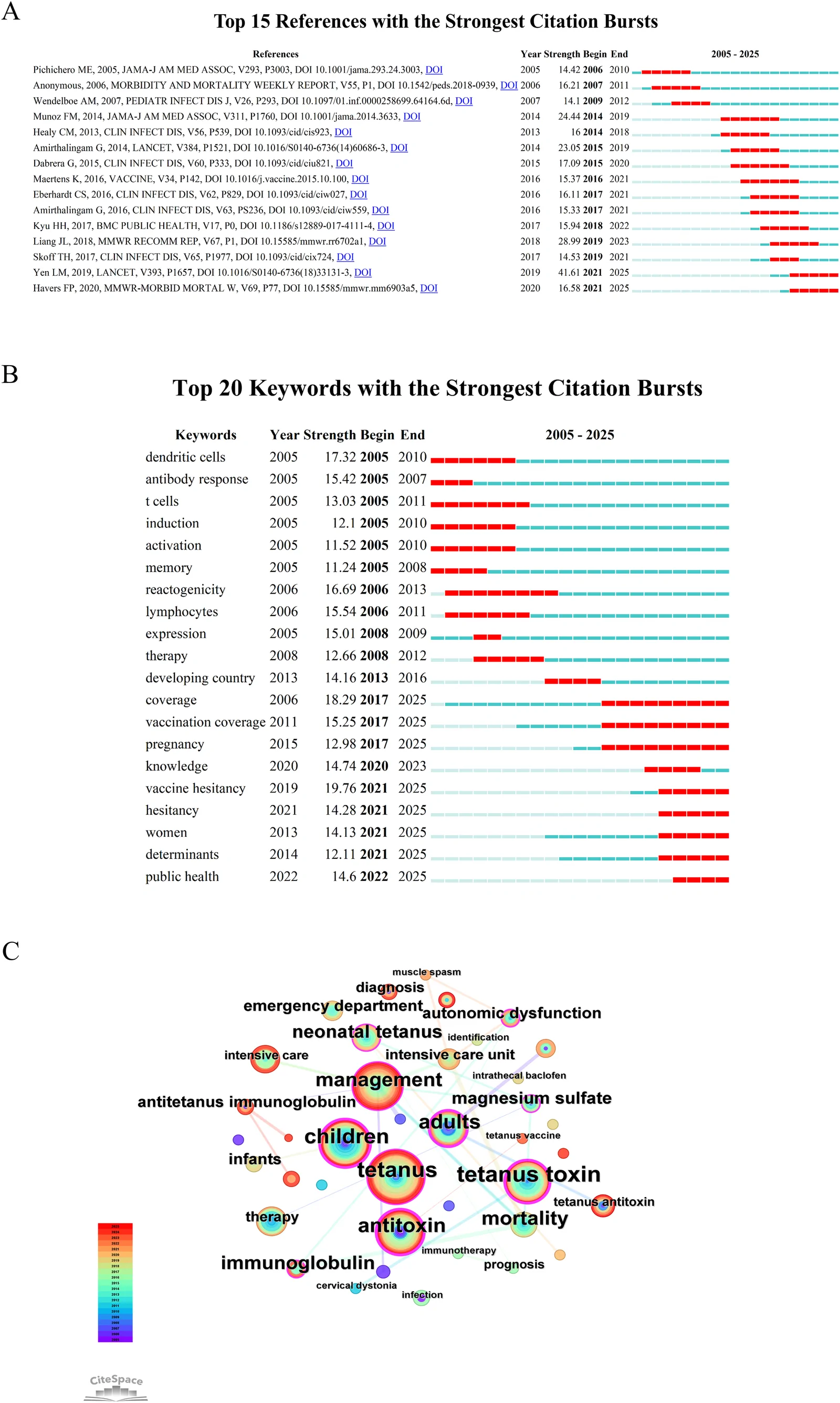
Research frontiers and clinical-management hotspots in tetanus-related research. References with the strongest citation bursts(Kleinberg algorithm: γ = 1.0, Minimum Duration = 2). B. Keywords with the strongest citation bursts(Kleinberg algorithm: γ = 1.0, Minimum Duration = 2). C. Keyword network of the clinical-management subset.

Since 2017, multiple implementation-relevant keywords have exhibited sustained occurrence bursts extending to 2025. Recent burst terms included coverage (strength = 18.29, 2017–2025), vaccination coverage (strength = 15.25, 2017–2025), pregnancy (strength = 12.98, 2017–2025), women (strength = 14.13, 2021–2025), determinants (strength = 12.11, 2021–2025), vaccine hesitancy (strength = 19.76, 2021–2025), and public health (strength = 14.60, 2022–2025). These time-resolved burst signals demonstrate growing publication-level interest in practical immunization delivery, maternal vaccination, barriers to vaccine uptake, and social-behavioral factors shaping immunization performance.

These keyword-burst patterns indicate that published tetanus-related literature has placed increasing emphasis on real-world immunization strategies, while basic immunological and toxin-focused research remained continuously active throughout the observation period. While effective tetanus toxoid vaccines are well-established, recent burst signatures highlight recurrent operational challenges reported in scholarly publications: sustaining vaccination uptake, improving adult-booster compliance, scaling maternal immunization, resolving population-level coverage gaps, and mitigating vaccine hesitancy. It is worth noting that this pattern only describes trends within bibliographic records; Therefore, recent burst signatures indicate growing publication-level attention to implementation-related issues, including vaccination uptake, maternal immunization, coverage gaps, and vaccine hesitancy. These signals do not imply that mechanistic, clinical, or other research domains have declined in importance, nor do they establish a corresponding change in real-world research or policy priorities.

### 3.5 Clinical-management subdomain

Automated term screening followed by manual verification identified 675 publications for inclusion in the supplementary clinical-treatment subset. Because the overall tetanus literature was dominated by vaccination- and prevention-related topics, a focused keyword co-occurrence analysis of this subset was conducted to characterize treatment-related themes. As shown in Fig. 4C, the clinical-management subdomain displayed a distinct thematic structure.

Core terms in this subdomain included management, children, adults, neonatal tetanus, antitoxin, immunoglobulin, intensive care, autonomic dysfunction, magnesium sulfate, and mortality. These keywords indicate that the clinical-treatment literature addressed severe disease management, antitoxin or immunoglobulin therapy, intensive care support, autonomic instability, neonatal and adult tetanus, and mortality.

Clinical-management topics were less prominent than vaccination- and prevention-related topics in the overall bibliometric analyses. Nevertheless, the supplementary analysis showed that clinical treatment constituted a distinct component of the broader tetanus literature. The clinical-management map therefore complements the prevention-focused findings by characterizing the principal topics represented in this subset.

## 4 Discussion

### 4.1 Principal findings

This bibliometric study mapped the global landscape of tetanus-related research from 2005 to 2025. Based on 8307 publications, we found that annual research output remained relatively stable over the past two decades, with a modest increase after 2010. Within the analyzed English-language dataset, the United States was the leading contributor, followed by the United Kingdom, India, Germany, and China. Major research contributors included public health agencies, universities, research institutes, and vaccine-related organizations, while Vaccine was the dominant publication venue. The thematic structure showed that emerging research frontiers placed increasing emphasis on vaccine strategies, maternal immunization, adult boosters, seroprotection, vaccination coverage, vaccine hesitancy, and public-health implementation, while toxin-biology and cellular-immunology-related themes remained ongoing research components. This pattern is broadly consistent with the current emphasis on life-course immunization, booster vaccination, maternal immunization, and sustained tetanus protection [1], [5], [14], [15]. Although clinical management represented a smaller proportion of the literature, it formed a distinct subdomain centered on management, antitoxin, immunoglobulin, intensive care, autonomic dysfunction, neonatal tetanus, adults, children, and mortality. This finding is also consistent with previous clinical and treatment-oriented literature, including studies on intensive care management and magnesium sulfate use in severe tetanus [3], [16].

Compared with the recent bibliometric and integrative review focusing specifically on tetanus vaccines and tetanus immunoglobulins [11], our study covered the broader tetanus research field, including toxin biology, immunology, clinical management, prophylaxis, maternal and neonatal tetanus, population immunity, and public health implementation. Therefore, the present analysis provides a wider view of the intellectual structure and thematic evolution of tetanus-related research.

### 4.2 Global productivity and collaboration

The predominance of the United States is consistent with its long-standing role in vaccine research, infectious disease surveillance, immunization policy, and global public health. Institutions such as the Centers for Disease Control and Prevention have contributed substantially to tetanus surveillance, vaccine recommendations, wound prophylaxis guidance, and immunization program evaluation [14], [15]. The strong contribution of the United Kingdom, including institutions such as the London School of Hygiene & Tropical Medicine and Oxford, reflects its influence in global health, epidemiology, vaccine evaluation, and infectious disease research.

The high productivity of India and China is also notable. These countries have large populations, extensive immunization programs, and important public health interests in vaccine-preventable diseases. India's contribution may be particularly related to maternal and neonatal tetanus prevention, immunization coverage, and public health implementation, while China's growing output may reflect increasing attention to vaccination strategies, population immunity, and infectious disease prevention. These trends are aligned with global efforts to strengthen routine immunization systems and broaden protection against vaccine-preventable diseases [5], [6].

International collaboration patterns provide an additional perspective. Belgium, France, the United Kingdom, and Canada showed high proportions of multiple-country publications, which may be associated with multinational vaccine trials, international public health programs, vaccine manufacturers, and collaborations involving organizations such as WHO and UNICEF. However, a high MCP% indicates a high proportion of internationally co-authored publications, not necessarily a large absolute volume of international collaboration, research leadership, or alignment with disease burden. Multinational vaccine manufacturers such as GSK also act as important collaborative hubs within this global network. Conversely, some low-income countries may experience a greater tetanus burden but contribute fewer indexed publications, suggesting an imbalance between disease burden, research capacity, and bibliographic visibility. Strengthening research capacity and data visibility in high-burden settings remains important for future tetanus prevention and control [5], [6].

### 4.3 Growing prominence of applied immunization themes among emerging research fronts

The knowledge structure indicates that emerging research frontiers have placed increasing emphasis on applied immunization strategies, although mechanistic and immunological investigations remained an important component of tetanus research across the whole period. Earlier research themes, including tetanus neurotoxin, dendritic cells, T cells, antibody response, immune activation, and memory, reflected interest in the biological mechanisms of tetanus pathogenesis and vaccine-induced protection. These studies provided the scientific basis for understanding neurotoxicity, immune response, and the protective role of antitoxin antibodies [3], [7].

As tetanus toxoid vaccine platforms and immunization programs matured, research attention increasingly shifted toward vaccine schedules, combination vaccines, toxoid conjugate vaccines, booster doses, antibody persistence, reactogenicity, and safety. The prominence of infant combination vaccines is expected because tetanus antigens are commonly delivered as part of diphtheria-tetanus-pertussis-containing vaccines in childhood immunization programs [1], [14]. Similarly, the visibility of adult boosters reflects the importance of maintaining protective immunity beyond childhood, especially because immunity may decline over time and natural infection does not reliably induce protection [1], [4], [14], [15].

Maternal immunization also emerged as a major and persistent theme. This reflects the importance of tetanus toxoid-containing vaccines in preventing maternal and neonatal tetanus, especially in settings where clean delivery practices and antenatal care access may be limited [2], [5]. Collectively, keyword-burst data (Fig. 4B) show that implementation-focused themes have gained growing prominence in tetanus-related publications from 2017 to 2025, whereas mechanistic research remained ongoing across the whole study period. This bibliometric finding describes publication trends rather than a definitive shift in real-world research priorities.

### 4.4 Growing attention to public health implementation

The recent burst terms identified in this study suggest that public health implementation has become a central frontier in tetanus research. Keywords such as coverage, vaccination coverage, pregnancy, women, knowledge, determinants, vaccine hesitancy, and public health indicate that current research increasingly focuses on the social, behavioral, and health-system factors that determine vaccine uptake. This is highly consistent with the broader vaccine literature, in which vaccine hesitancy, public confidence, misinformation, and access-related barriers have become major determinants of immunization performance [17], [18], [19]. At the same time, global monitoring reports continue to emphasize the importance of improving and sustaining vaccination coverage [20], [21].

This shift is highly relevant because tetanus is preventable but not eradicable through herd immunity. Protection depends on individual immunity acquired through vaccination or appropriate post-exposure prophylaxis [1], [4]. Therefore, sustained high coverage, accurate vaccination records, timely boosters, and effective wound management remain essential. Declining immunity among adults and older populations, incomplete vaccination histories, and missed booster opportunities may create immunity gaps even in countries with strong childhood immunization programs [1], [14], [15].

Maternal and neonatal tetanus prevention remains closely linked to antenatal care, clean delivery, women's access to health services, and maternal vaccination coverage [2], [5]. The emergence of pregnancy and women as recent research frontiers suggests continued attention to maternal immunization as both an individual and neonatal protection strategy. Evidence from pregnancy vaccination studies also supports the broader importance of maternal immunization programs for protecting mothers and infants. At the same time, vaccine hesitancy and knowledge-related barriers have become increasingly important. Even when vaccines are available, misinformation, low perceived risk, access barriers, and reduced trust in health systems may affect uptake [17], [18], [19]. In addition, disruptions to routine immunization during the COVID-19 pandemic further highlighted the vulnerability of vaccination systems and the need for resilient immunization services [22], [23]. Existing public-health evidence indicates that tetanus prevention involves not only vaccine availability but also communication, service accessibility, targeted outreach, and integration into life-course immunization programs.

### 4.5 Clinical management remains a distinct but less prominent research domain

Although vaccination- and prevention-related topics were more prominent in the overall bibliometric landscape, the supplementary clinical-treatment subset identified an important body of treatment-oriented literature. These results should not be interpreted as evidence of treatment efficacy, because bibliometric analysis cannot directly assess clinical effectiveness. Rather, they indicate the main topics around which clinical tetanus research has been organized.

The clinical relevance of this subdomain remains substantial. Once symptomatic tetanus occurs, patients may require high-intensity supportive care, including toxin neutralization, wound management, control of muscle spasms, respiratory support, and management of autonomic instability [3], [24]. Severe tetanus can be associated with prolonged hospitalization and high mortality, particularly where intensive care, mechanical ventilation, antitoxin or immunoglobulin, and trained clinical staff are limited [3], [24]. Previous clinical studies and reviews have examined the use of magnesium sulfate for severe tetanus, but treatment decisions remain dependent on clinical severity, local resources, and supportive care capacity [16]. More recently, new therapeutic approaches, including recombinant monoclonal antibodies against tetanus toxin, have also attracted attention and may influence future treatment strategies [25].

The relative underrepresentation of clinical-management topics in the overall bibliometric landscape may partly reflect the longstanding emphasis on vaccination and prevention in tetanus research. However, bibliometric data cannot determine the reasons for this pattern, and the clinical importance of treatment research should not be underestimated. Future studies are needed to improve standardized management pathways, evaluate resource-adapted intensive care strategies, optimize treatment of autonomic dysfunction, improve access to antitoxin or immunoglobulin, and assess outcomes in neonates, adults, older patients, and resource-limited settings. In emergency care, accurate tetanus risk assessment, appropriate post-exposure prophylaxis, and point-of-care assessment of tetanus immunity also remain important issues [26], [27]. Such work would complement prevention-focused research and help reduce mortality among patients who develop clinical tetanus.

### 4.6 Implications and limitations

This study suggests several practice-oriented implications for tetanus research and public health. These implications are informed by the bibliometric patterns observed in the present dataset and should be interpreted alongside evidence from the broader substantive literature.

First, sustained international research collaboration may be particularly valuable for tetanus prevention research in settings where disease burden remains substantial but indexed publication output is relatively limited. Our country-level analysis showed uneven patterns of research production and international collaboration, with relatively high proportions of international collaboration among several high-income European countries (Section 3.2). When considered alongside published estimates of tetanus burden, these findings suggest a possible geographical mismatch between indexed research activity and public health need in some settings. Cross-national partnerships could support local research capacity, facilitate the generation of context-specific evidence, and increase the representation of research from underrepresented high-burden regions. Together with strengthened surveillance and standardized reporting, such partnerships may also contribute to the identification of vulnerable populations and the monitoring of progress toward tetanus-control goals [28]. Second, maternal immunization, adult booster vaccination, catch-up strategies, vaccination coverage, and vaccine hesitancy may warrant continued research and public health attention. Our keyword-burst and thematic-structure analyses identified vaccination coverage, pregnancy-related topics, women, and vaccine hesitancy as recent or emerging areas of scholarly attention (3.3, 3.4). These findings indicate growing research interest in implementation-related vaccination issues, although bibliometric prominence alone does not establish intervention effectiveness or policy priority. Evidence from the substantive literature nevertheless suggests that appropriately designed interventions in these areas may help address population-level immunity gaps, strengthen maternal and neonatal tetanus protection, and reduce barriers to vaccine uptake [5], [6], [14], [15], [17], [18], [19], [20], [21]. Third, population-level seroprotection and immunity-gap assessments deserve continued investigation among older adults, migrants, individuals with incomplete vaccination histories, and other potentially vulnerable groups. Specific populations, including patients with chronic or diabetic foot wounds and people who inject drugs, may also benefit from more tailored research on vaccination and wound-prophylaxis strategies. This implication is consistent with our thematic mapping, which identified seroprotection and topics related to specific at-risk populations within the public health research domain (Section 3.3). Further studies involving these populations may help characterize immunity deficits, inform targeted vaccination and prophylaxis policies, and support the development of more appropriately tailored prevention strategies [7], [29], [30]. Fourth, clinical tetanus research may warrant greater attention to treatment accessibility, intensive care capacity, antitoxin or tetanus immunoglobulin availability, and management approaches that are feasible in resource-limited settings. In our clinical-subdomain analysis, clinical-management topics were relatively less prominent in the mapped literature (Section 3.5). This bibliometric pattern does not by itself demonstrate inadequate clinical care, but, when considered alongside the reported severity and mortality of symptomatic tetanus in resource-constrained settings, it suggests a need for further context-specific clinical research. Such research could inform the development of feasible treatment protocols, clarify barriers to essential therapies and intensive-care resources, and identify research gaps that may, in future work, help to optimize clinical management for tetanus patients [3], [16], [24], [25].

More broadly, this study illustrates the complementary role of longitudinal bibliometric mapping in evidence synthesis. By integrating publication trajectories, citation influence, collaboration networks, and keyword evolution, bibliometric approaches can characterize shifts in scholarly attention and identify emerging research fronts [31], [32]. However, bibliometric evidence reflects the production, dissemination, and organization of indexed literature rather than real-world disease incidence, population burden, or patient prognosis. Topic prominence therefore does not necessarily indicate research priority, intervention effectiveness, or public health policy demand. These outcomes require structured synthesis of primary empirical evidence, including burden- and prognosis-oriented studies and incidence-focused systematic reviews and meta-analyses [33], [34]. Accordingly, trends in tetanus publications should be interpreted alongside official surveillance data, epidemiological studies, and high-quality systematic reviews. Such triangulation helps distinguish publication visibility from actual public health needs and prevents keyword prominence or citation impact from being treated as direct proxies for disease burden, intervention effectiveness, or policy priority.

Several limitations should be acknowledged. First, relevant studies indexed outside WoSCC and Scopus may have been missed. Second, restricting the analysis to English-language publications may have underrepresented research from non-English-speaking regions and affected interpretation of the global research landscape. Third, citation indicators are subject to time lag and may underestimate recent publications. Fourth, author- and institution-level analyses may be affected by name ambiguity, affiliation changes, institutional variants, and inconsistent cited-reference metadata. Because dataset - wide disambiguation and standardization of these fields were not performed, residual node fragmentation or erroneous merging cannot be excluded—a common limitation when combining WoSCC and Scopus records. In addition, keyword analyses were constrained by cross-database heterogeneity in keyword metadata and the absence of synonym consolidation; synonymous or morphologically similar terms remained as separate nodes in co-word networks, potentially fragmenting thematic visualization. Fifth, CiteSpace and VOSviewer clustering results depend on algorithmic parameters and labeling methods; cluster labels should therefore be regarded as thematic approximations rather than definitive classifications [8], [9], [12], [13]. Sixth, bibliometric analysis identifies publication patterns, influential topics, collaboration structures, and emerging research fronts but cannot directly assess clinical or policy effectiveness, causal relationships, or changes in disease incidence and burden [8], [9], [10]. Publication output and keyword prominence may also be shaped by funding priorities, database coverage, indexing practices, international initiatives, and terminology changes. The observed trends should therefore be interpreted as shifts in scholarly attention rather than direct evidence of changes in tetanus epidemiology or public health importance. Finally, eligibility assessment was conducted by one researcher, precluding measurement of inter-reviewer agreement. Although predefined hierarchical exclusion criteria and repeated review of uncertain records were used to reduce subjectivity, reviewer-dependent judgment and potential misclassification cannot be excluded.

## 5 Conclusion

Overall, this study shows that, while toxin biology and immune-response research remained important components of the tetanus literature, recent research fronts increasingly emphasized vaccine strategies, maternal and adult immunization, vaccination coverage, vaccine hesitancy, and public health implementation. Clinical-management topics were less prominent in the overall bibliometric landscape but constituted a distinct research subdomain. Future tetanus control will require sustained immunization systems, strengthened global collaboration, targeted protection of vulnerable populations, improved wound prophylaxis, and optimized clinical care for severe tetanus. These bibliometric findings reflect publication-level trends and should be interpreted in conjunction with clinical, epidemiological, and surveillance evidence.

The following are the supplementary data related to this article.

**Supplementary Fig S1:**
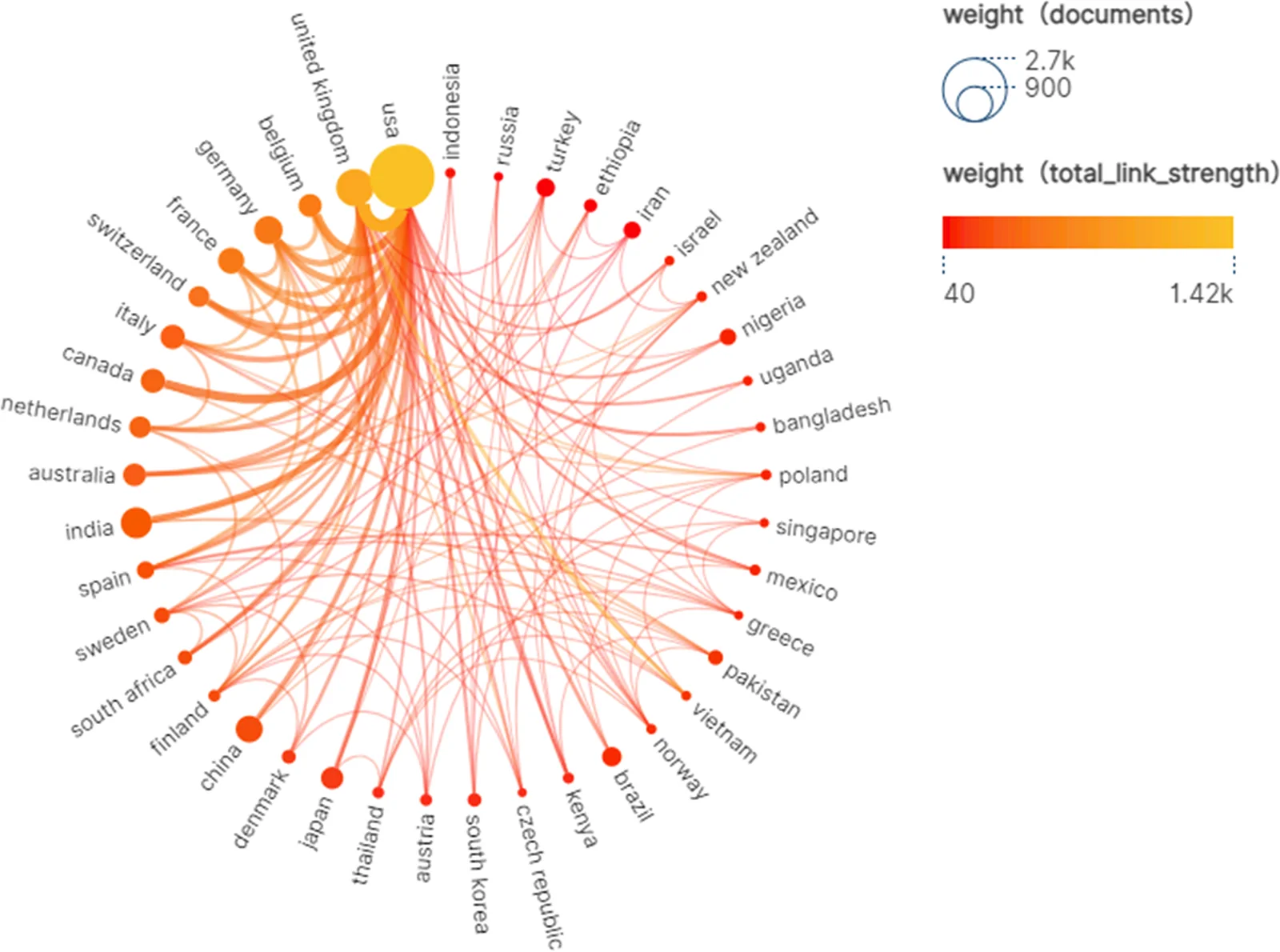
Country collaboration network based on total link strength(minimum country occurrence = 50).

**Supplementary Fig S2:**
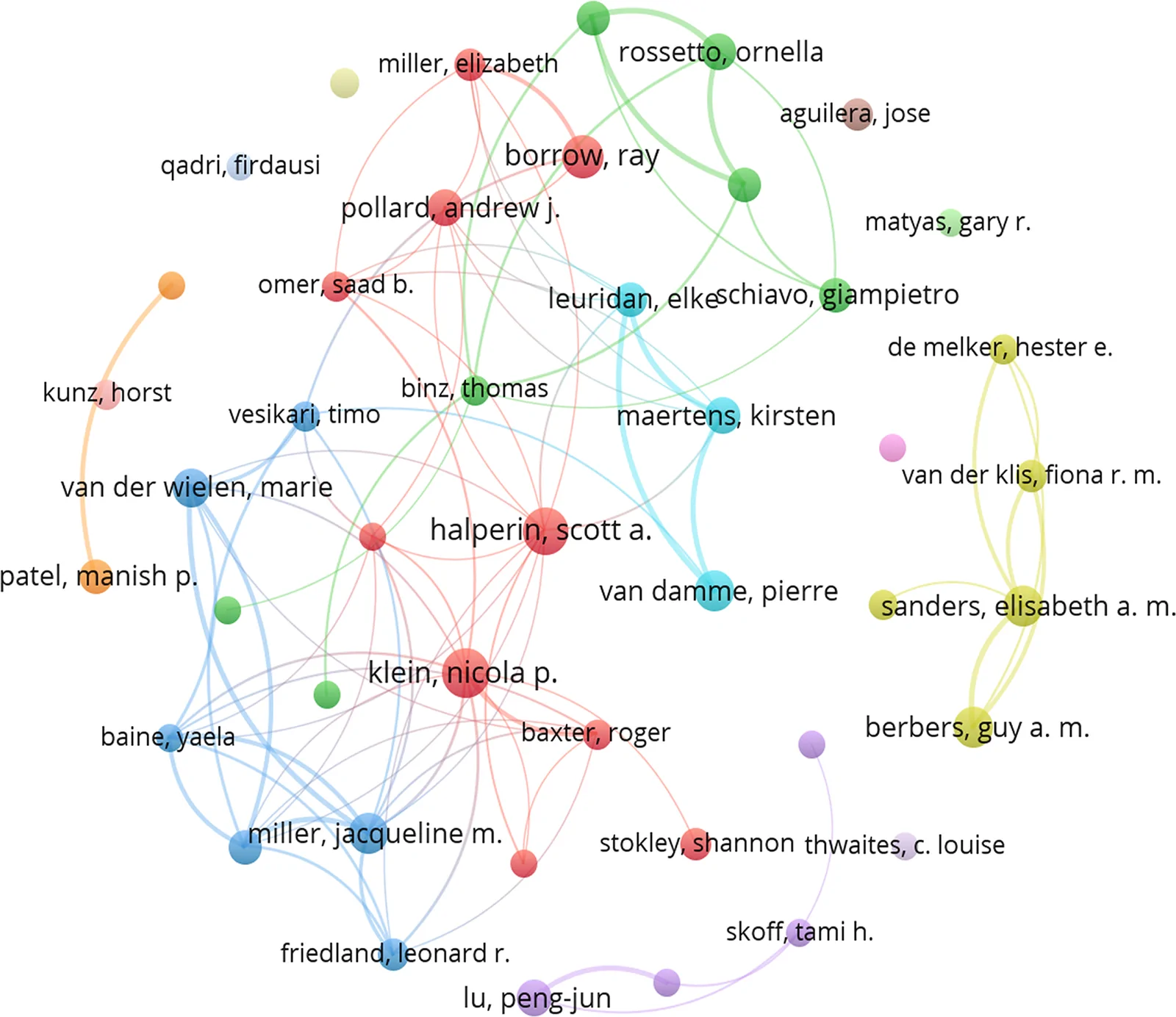
Author collaboration network(minimum documents per author = 14).

**Supplementary Fig S3:**
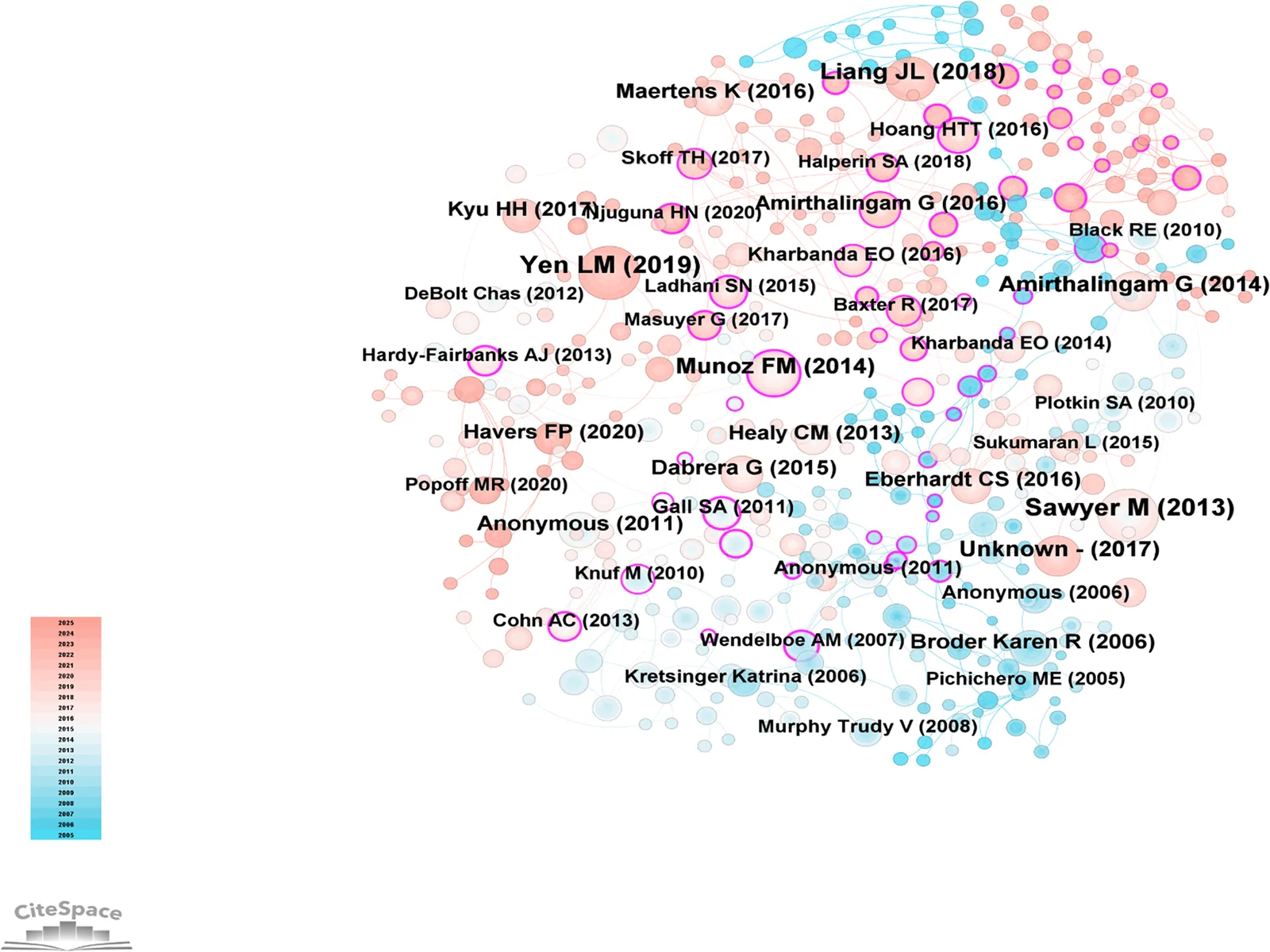
Co-cited reference network.

**Supplementary Fig S4:**
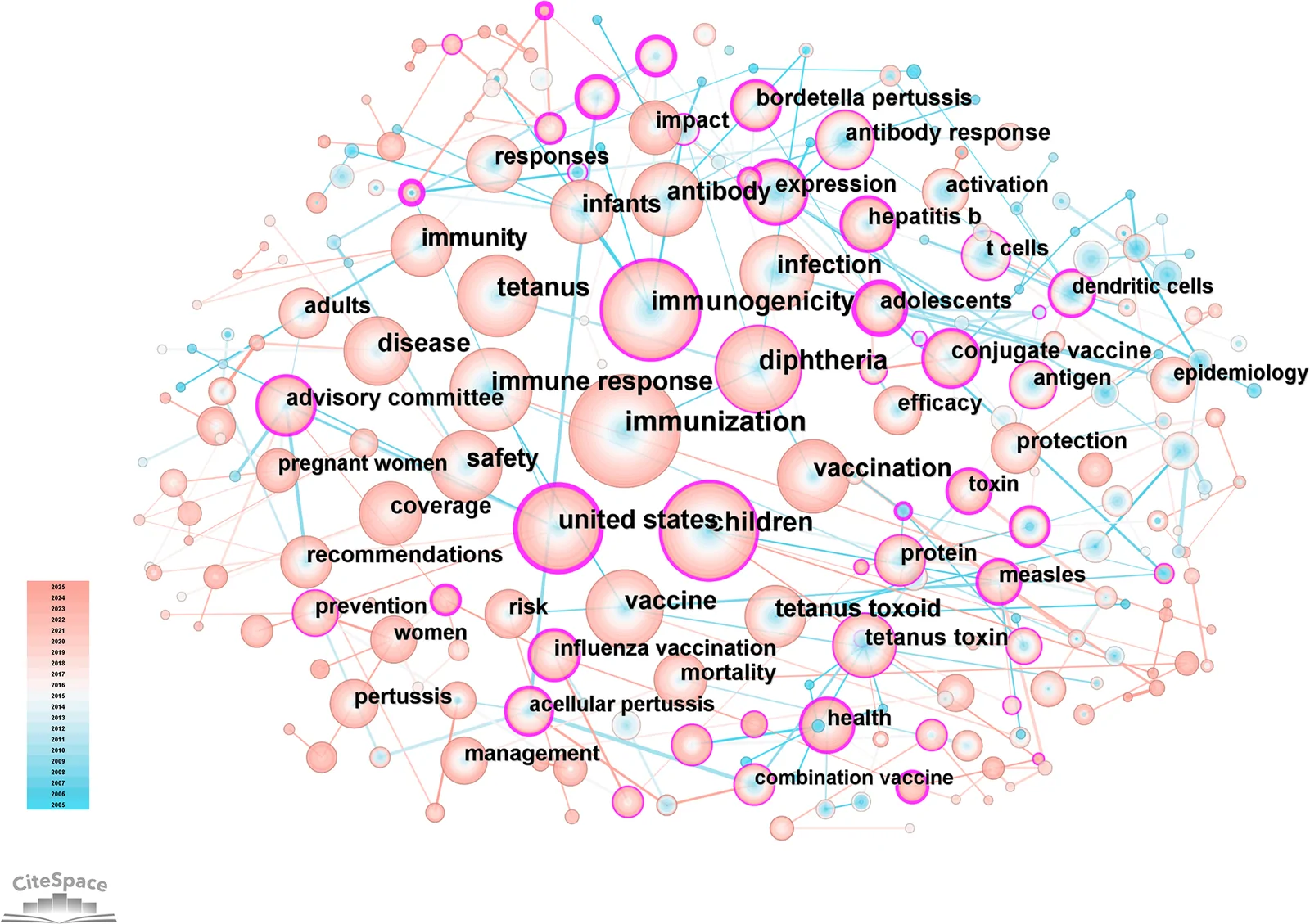
Keyword co-occurrence network.

**Supplementary Fig S5:**
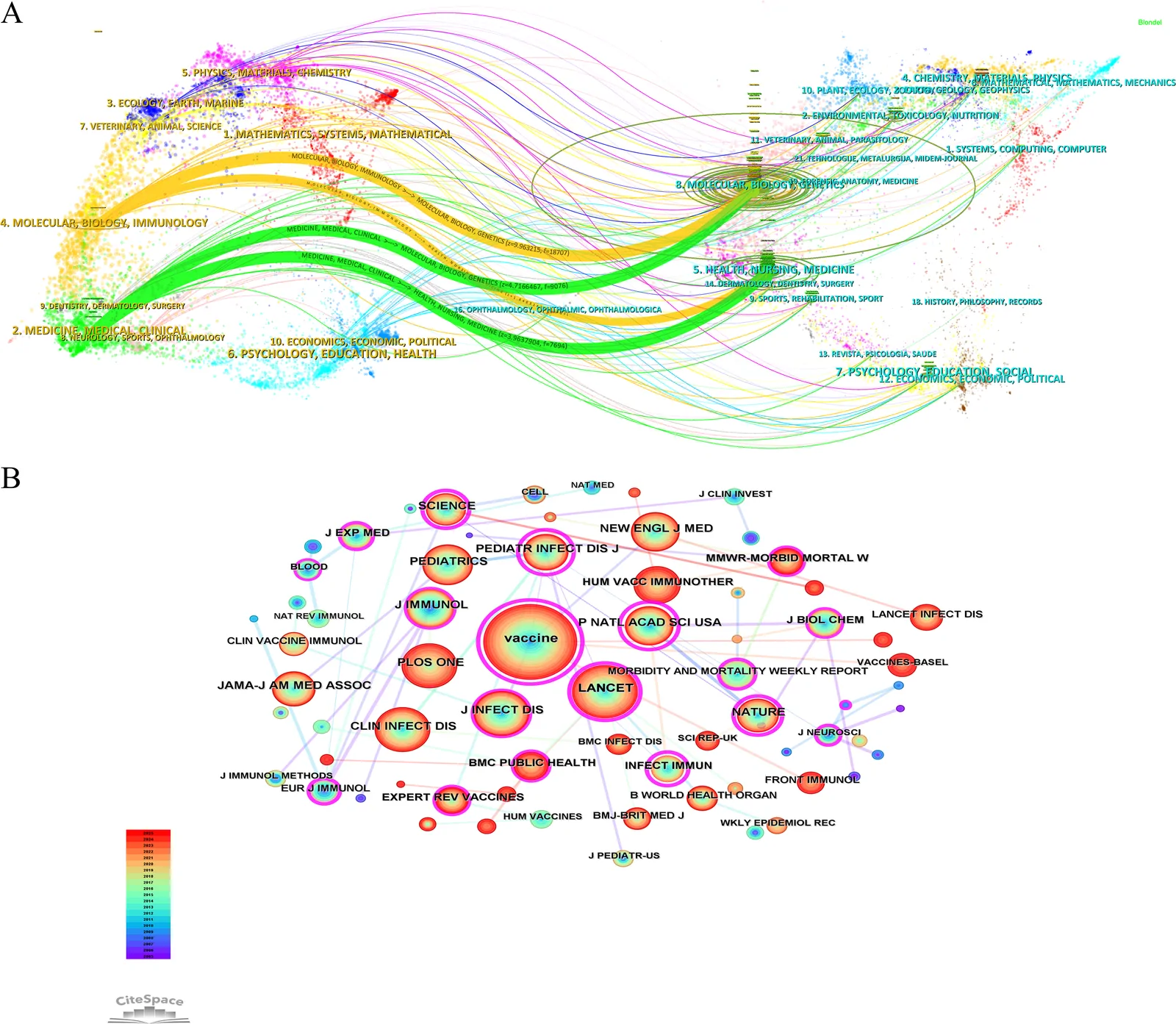
Journal-level intellectual structure and disciplinary citation patterns in tetanus-related research. A. Dual-map overlay of journals. B. Co-cited journal network(Top *N* = 30 per time-slice).

**Supplementary Fig S6:**
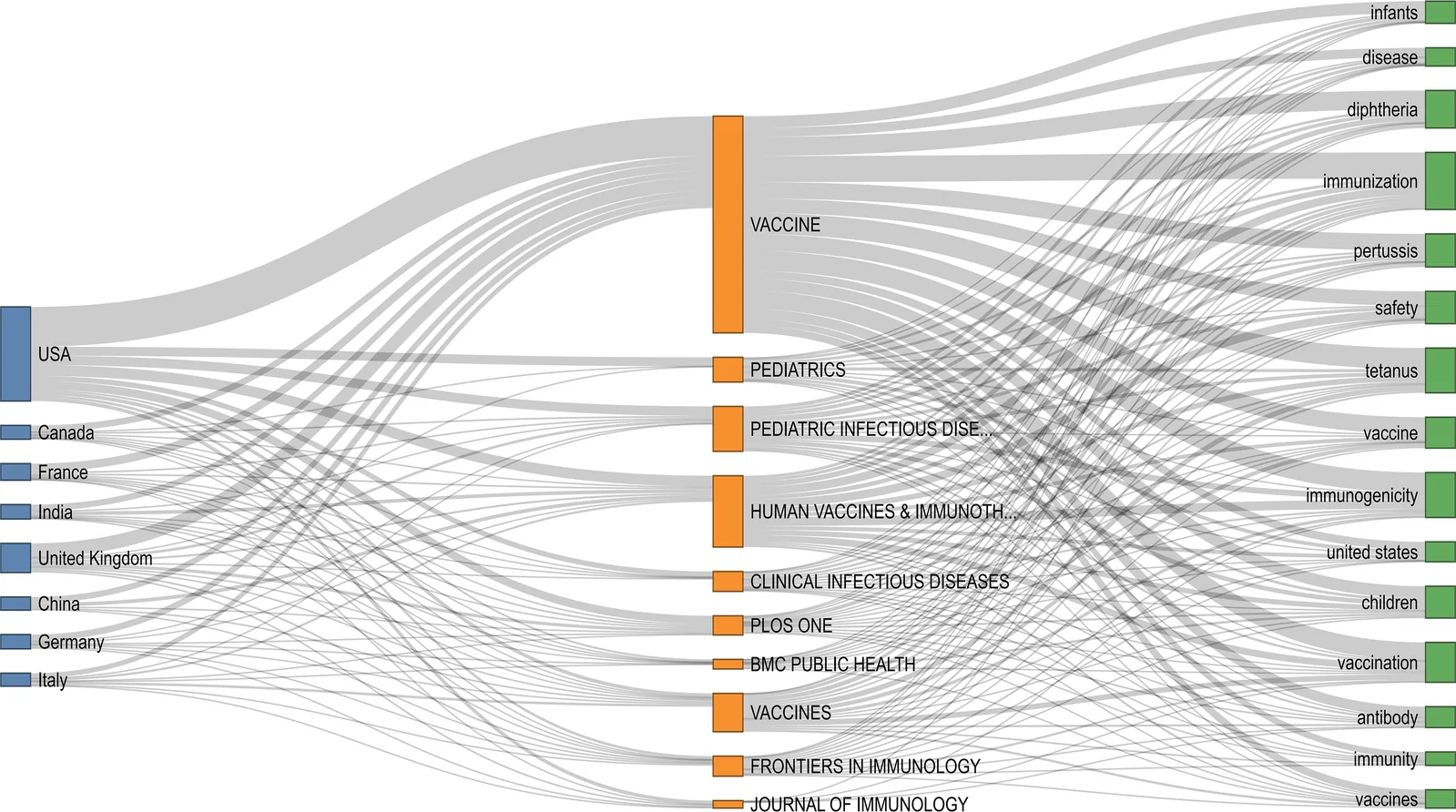
Three-field plot linking countries, journals, and keywords in tetanus-related research.

Supplementary Table S1 Standardized replacement table for country and region names.

## CRediT authorship contribution statement

**Dandan Zhou:** Writing – original draft, Visualization, Methodology, Investigation, Conceptualization. **Ji Zhang:** Writing – original draft, Methodology, Investigation, Conceptualization. **Ke Ma:** Investigation. **Tianjing Sun:** Investigation. **Fangfang Deng:** Investigation. **Shengping Wu:** Investigation. **Han Hu:** Visualization. **Haizhen Duan:** Investigation. **Tianxi Zhang:** Investigation. **Anyong Yu:** Writing – review & editing, Supervision. **Xuheng Jiang:** Writing – review & editing, Supervision.

## Consent for publication

All authors have read and approved the final version of the manuscript and consent to its publication in this journal.

## Ethical approval and consent to participate

As this study is a bibliometric analysis based on previously published data, no human participants, animals, or clinical samples were directly involved. Therefore, ethical approval was not required.

## Funding

This work was supported by the 10.13039/501100001809National Natural Science Foundation of China (82260254); the High-level innovative talents of Guizhou Province (GCC [2023]081); Guizhou Province Clinical Research Center for Emergency Medicine (No. LCZX[2025]004); and the Guizhou Province Traditional Chinese Medicine, Ethnic Medicine Science and Technology Research Special Project (QZYY-2025-033); Guizhou Provincial Science and Technology Plan Project (LC [2026] 003).

## Declaration of competing interest

The authors declare that they have no known competing financial interests or personal relationships that could have appeared to influence the work reported in this paper.

## Data Availability

Datasets are available upon request from the corresponding author.
