# Supplementary material for: Global trends and knowledge structure of tetanus research from 2005 to 2025: A bibliometric analysis based on web of science Core Collection and Scopus
Source: One Health. 2026 Sep 20;23:101590. doi: 10.1016/j.onehlt.2026.101590 (PMC13634260; doi:10.1016/j.onehlt.2026.101590)
Supplement: Supplementary file 1 [file mmc7.docx]

| label | replace by |
| --- | --- |
| usa | USA |
| u s a | USA |
| united states | USA |
| united states of america | USA |
| us | USA |
| peoples r china | China |
| people s r china | China |
| peoples republic of china | China |
| peoples republic china | China |
| pr china | China |
| p r china | China |
| china mainland | China |
| mainland china | China |
| china | China |
| taiwan | China |
| england | United Kingdom |
| scotland | United Kingdom |
| wales | United Kingdom |
| northern ireland | United Kingdom |
| north ireland | United Kingdom |
| uk | United Kingdom |
| u k | United Kingdom |
| united kingdom | United Kingdom |
| great britain | United Kingdom |
| britain | United Kingdom |
| south korea | South Korea |
| republic of korea | South Korea |
| korea | South Korea |
| korea south | South Korea |
| iran | Iran |
| islamic republic of iran | Iran |
| russia | Russia |
| russian federation | Russia |
| germany | Germany |
| deutschland | Germany |
| france | France |
| italy | Italy |
| canada | Canada |
| japan | Japan |
| australia | Australia |
| belgium | Belgium |
| india | India |
| spain | Spain |
| netherlands | Netherlands |
| holland | Netherlands |
| switzerland | Switzerland |
| sweden | Sweden |
| denmark | Denmark |
| norway | Norway |
| finland | Finland |
| poland | Poland |
| turkey | Turkey |
| turkiye | Turkey |
| portugal | Portugal |
| mexico | Mexico |
| brazil | Brazil |
| south africa | South Africa |
| singapore | Singapore |
| malaysia | Malaysia |
